# *Mycobacterium neoaurum* Bloodstream Infection Associated with a Totally Implanted Subclavian Port in an Adult with Diabetes and History of Colon Cancer

**DOI:** 10.1155/2020/8878069

**Published:** 2020-12-31

**Authors:** Jack E. Moseley Jr., Sharanjeet K. Thind

**Affiliations:** ^1^Oklahoma Department of Veterans Affairs, Medical Director-Sulphur Center, 344 E Fairlane Avenue Sulphur, Oklahoma City 73086, OK, USA; ^2^Section of Infectious Diseases, Medical Service, Oklahoma City VA Medical Center, Department of Medicine, Oklahoma University Health Sciences Center, 921 NE 13th Street, Oklahoma City 73104, OK, USA

## Abstract

*Background*. *Mycobacterium neoaurum* is a rapidly growing nontuberculosis mycobacterium (NTM) that was first isolated from soil in 1972 and is ubiquitous in soil, water, and dust. The first reported case of human infection by *M. neoaurum* was published in 1988, presenting as a Hickman catheter-related bacteremia in a patient with ovarian cancer. *M. neoaurum* has since been recognized as a source of predominantly opportunistic bloodstream infections in immunocompromised hosts. We report the case of an adult diabetic male with *M. neoaurum* bloodstream infection secondary to an infected venous-access port that had been implanted nearly six years prior for temporary chemotherapy. *Case Presentation*. A 66-year-old male with schizophrenia, type 2 diabetes mellitus, and a history of excision and chemotherapy to treat adenocarcinoma of the colon 6 years prior, presented with fever and behavioral changes. He was found to have a *M. neoaurum* bloodstream infection secondary to his implanted subclavian port. Multiple preoperative blood cultures, as well as the removed catheter tip culture, were positive for *M. neoaurum*. The patient's condition improved to near premorbid levels after port removal and 6 weeks of targeted antimicrobial therapy. *Discussion and Conclusions*. Bloodstream infections due to rapidly growing NTM, such as *M. neoaurum*, have been infrequently reported; however, improved isolation and identification techniques based on genomic testing are resulting in a more in-depth recognition of these widely scattered environmental microbes in human infections. Nonetheless, lengthy identification and susceptibility processes remain a diagnostic and treatment barrier. Patients such as ours who have a history of malignancy and an indwelling foreign body have most often been reported as acquiring *M. neoaurum* bacteremia. Fortunately, device removal and appropriate antimicrobial therapy guided by susceptibility data is often enough to manage these atypical mycobacterial infections.

## 1. Introduction

*Mycobacteriumneoaurum* is an aerobic acid-fast bacterium that is one of many nontuberculosis mycobacteria (NTM) species. *M. neoaurum* is categorized as a rapidly growing pigmented mycobacterium because it produces colony growth within a week with a typical gold (aurum) color. *M. neoaurum* was isolated from soil in 1972 and has been described as ubiquitous in soil, water, and dust but an infrequent source of human infection. Infections are typically bacteremia in immunocompromised hosts who have long-term central venous catheters or implanted foreign bodies such as pacemakers, prosthetic heart valves, and arteriovenous shunts [1, 2]. The following report describes the presentation, diagnosis, and treatment of unsuspected *M. neoaurum* bacteremia in an adult male with a totally implanted subclavian port that had been left in place for nearly 6 years after completing chemotherapy for colon cancer. He presented with no signs of recurrent cancer or immune suppression.

## 2. Case Presentation

A 66-year-old male with schizophrenia, moderate cognitive impairment, and type 2 diabetes mellitus underwent hemicolectomy for adenocarcinoma of the colon, after which he received several months of chemotherapy through a totally implanted right subclavian vascular access port and catheter. This device remained in place thereafter and he was clinically well during the intervening 6 years before presentation. He lived in a residential long-term care (LTC) facility managed by registered nurses with physician backup around the clock. His port and catheter device was sterilely flushed with normal saline and heparin every 60 days by a nurse manager during this interval to maintain patency although the device was no longer being used. There had been no known port-related infections. The patient had no other indwelling catheters, or implanted devices. He was edentulous and had no prosthetic devices. He had no history of renal or urinary tract disease. He had had a single seizure 23 years previously followed by a normal EEG. He had a long history of self-abusive head slapping which averaged several times each week, resulting in a traumatic cataract and loss of sight in his right eye. He was independently ambulatory and took care of his own activities of daily living. His current presentation began with back and leg pains and onset of a shuffling gait a month after his previous 60-day port flush, which had taken place without complications. A nurse and another caregiver at his LTC facility assisted him after lunch one day to walk steadily back several meters from the dining area to his bedroom. He had had no tripping or falls but his mentation seemed a little slow and he wanted to sleep. Before going to bed, his vital signs and finger-stick blood glucose were checked. He was afebrile, hemodynamically stable, and euglycemic. Fourteen hours later, he was discovered kneeling on the floor beside his bed, unable to follow commands or explain how he was feeling. His temperature was 39.2 degrees Celsius, pulse 136 beats/minute, respirations 54 breaths/minute, blood pressure 137/65 mm Hg, and oxygen saturation 89% on room air. His finger-stick blood glucose was 328 mg/dL. A mechanical body lift was used to get him off the floor. He was transferred to the community hospital emergency department (ED) where he exhibited no bruising or signs of pain, trauma, breathlessness, rigidity, or shaking. The skin covering his implanted chest port and subclavian catheter appeared normal, without redness, warmth, swelling, or tenderness. His heart was in sinus rhythm; his electrocardiogram (ECG) showed no ischemic findings. His leukocyte count was 9.3 × 10^9^/L with a normal differential and hemoglobin of 15.2 g/dL. His initial troponin was mildly elevated but serial troponin values that followed were normal. His serum creatinine level was 1.36 mg/dL (reference range, 0.70–1.25 md/dL); however, his blood creatine kinase (CK) level, previously normal, was elevated at 529 U/L and later peaked at 23 961 U/L (reference range, 0–200). His electrolytes, alanine aminotransferase (ALT), and aspartate transaminase (AST) levels were normal. His hemoglobin A1c was 9.4%. Urine drug screen was negative, and his influenza A and B tests were negative. His urinalysis showed amber color urine, 3–5 RBC/hpf, rare epithelial cells, 1 + mucous, and 0 WBC/hpf. A noncontrast computed tomography (CT) scan of his brain revealed no midline shift, mass effect, herniation, or territorial infarct. The calvarium and scalp were normal. Chest radiographs identified his port and catheter but no infiltrates, nodules, pneumothorax, or abnormal findings (his routine annual purified protein derivative (PPD) had been done one week prior and had been negative). Due to his severe rhabdomyolysis, the patient was transferred to a regional tertiary medical center. Blood cultures from that admission were reported as negative on hospital day 4. He was discharged after 9 inpatient days, off all antibiotics, and no source of fever had been discovered. His gait was his usual. His CK level was 441 U/L and soon returned and remained within the normal reference range.

During the first 4 months after his discharge from the medical center, he had some intermittent gait disturbances with loss of balance at his LTC facility but no known falls. In the 4^th^ month, he had 2 days of fever (maximum 38.6 degrees Celsius) but no lab work was obtained, and he received no antibiotics. He complained of headache and back and leg pains. His nurses noted a slightly shuffling gait from time to time. In the fifth month, he developed a left-sided Bell's palsy 6 days after a routine sterile port flush with saline and heparin. He complained of a left earache. He was afebrile but was also noted to be having increased delusions and audiovisual hallucinations. His otoscopic exam was normal and revealed no vesicles or sores. He was alert, cooperative, and followed verbal instructions to sit in a chair and lift himself up to a standing position, which he did immediately without use of armrests or other assistance. He demonstrated that he could walk back and forth across the room slowly but steadily and sit back down. A left foot drag that was not known previously was noted by a physician who was examining him. He was started on valacyclovir and prednisone. The next day, the color of the patient's skin appeared sallow and he admitted that he was not feeling well. He was transferred to the community hospital ED where his vital signs were normal including oxygen saturation of 94% on room air. He was incontinent of urine but had no cough, dyspnea, icterus, or rash. He was noted to have a left-sided Bell's palsy, and his ear examination was normal. The skin over his chest port appeared normal. A repeat noncontrast CT scan of his brain and skull showed no interval changes and no mastoid pathology. His leukocyte count and hemoglobin were normal; his CK was normal (45 U/L); and his creatinine, electrolytes, bilirubin, and transaminases were normal. His influenza tests were negative and his nasopharyngeal polymerase chain reaction (PCR) for Severe Acute Respiratory Syndrome Coronavirus 2 (SARS CoV-2) test came back later as negative. He was given intravenous fluids, started on levofloxacin for possible urinary tract infection, and discharged back to his LTC facility.

Within 48 hours, he spiked a fever to 39.3 degrees Celsius, and his pulse was 120 beats per minute. His oxygen saturation on room air was 96% and he had no cough. His gait became unsteady, so he was transferred back to the community hospital ED, where his leukocyte count was 8.3 × 10^9^/L, creatinine was 1.26 mg/dL, and urinalysis showed 10 WBC/hpf and 1 + bacteriuria. His blood pressure was stable. A third noncontrast CT scan of the brain and head demonstrated no acute changes. His CT scan with contrast of the chest revealed a 3.3 cm left-sided thyroid nodule; the port with its tip located in the right superior vena cava; no focal consolidation, effusion, or pneumothorax; normal heart size without pericardial effusion; normal thoracic aorta; moderate circumferential esophageal wall thickening; and no osseous injury or axillary adenopathy. His CT scan with contrast of the abdomen and pelvis revealed normal abdominal aorta and vascular tree; no focal liver, gallbladder, pancreatic, or splenic lesions; and normal adrenals, kidneys, and bladder. A right hemicolectomy was noted, and there was no sign of free air. He was transferred from there to a regional tertiary medical center for possible sepsis due to suspected urinary tract infection. Two sets of blood cultures were obtained, he was started on cefepime, and, over a few days, his condition improved. His urine culture came back negative. Five days after admission, an aerobic bottle from one of the two sets of blood cultures taken at admission turned positive with gram-positive cocci in chains; this was believed on that day to most likely be a contaminant. . The following day, the isolate was believed to be a Kinyoun-stain positive gram variable rod. The Oklahoma State Department of Health Laboratory utilized matrix-assisted laser desorption ionization-time of flight mass spectrometry (MALDI-TOF MS) to identify the isolate as *Mycobacterium neoaurum*. The colony characteristics of this and future blood culture isolates from our patient were consistent with *M. neoaurum*. A sample of the initial isolate was sent to the Advanced Diagnostics Mycobacterium Laboratory at National Jewish Health in Denver, Colorado, and reidentified as *M. neoaurum*, by rpoB gene sequencing. A month-long process of antibiotic susceptibility testing (AST) was started, using a Clinical and Laboratory Standards- (CLSI-) aligned method of broth microdilution minimum inhibitory concentration (MIC) tests.

In the interim, our patient finished a week of cefepime and had been discharged from the medical center. He was asymptomatic. Due to the isolation of *Mycobacterium neoaurum* from only 1 of the 2 sets of blood cultures (1 bottle) from his medical center admission, repeat blood cultures were drawn at his LTC facility 10 days after antibiotics had been stopped. One set of blood cultures was obtained from a peripheral arm vein and the other set drawn from his central line port. The patient looked and felt well at the time his blood was taken but about 15 hours later, he was incontinent of urine and had a fever of 39.1 degrees Celsius. He was also pounding his head with his fist and trying to throw himself out of bed. He did not answer questions, would not stand on his own, and slumped over if sat up. He was transferred again to the community hospital ED, where he had an unremarkable CT scan of the brain and an unremarkable chest radiograph and lab studies, including a normal leukocyte count. The skin over his port appeared normal, without erythema, swelling, and tenderness. He had no heart murmur and no lymphadenopathy. He was hydrated and observed in the ED while being started on levofloxacin for possible urinary tract infection. He became alert, afebrile, and ambulatory and wanted to return to the LTC facility. He was discharged to the LTC facility and doxycycline was added the next day to cover for possible *M. neoaurum* bacteremia. Two days later, both aerobic blood culture samples drawn from his arm and from his port before he was sent to the ED turned positive with a microorganism later identified as *M. neoaurum.*

The patient remained stable on dual antibiotic therapy and was admitted within 48 hours to the tertiary medical center and had surgical removal of his port and subclavian catheter. His central and peripheral venous blood was cultured the day before surgery and he was started on imipenem in addition to doxycycline and levofloxacin. The tip of his subclavian catheter was sent for culture and grew *M. neoaurum*. Three of the 5 peripheral blood cultures drawn the day before surgery were positive for *M. neoaurum* including 3 lyses centrifugation blood cultures. Blood drawn the day before surgery from the patient's central line port was also positive for *M. neoaurum*. Postoperative blood cultures drawn in the hospital 4 days after the removal of the port and subclavian catheter were negative.

The patient was discharged to his LTC facility on hospital day 6 and was continued on the combination of imipenem, levofloxacin, and doxycycline for about 4 weeks. He pulled out his peripherally inserted central catheter (PICC) line after 4 weeks of therapy, at which time we had just received the antibiotic susceptibility report with minimum inhibitory concentrations (MIC) from the National Jewish Health Mycobacteriology Laboratory in Denver, as follows:

  Amikacin MIC: ≤8mcg/mL = S
  Cefoxitin MIC: ≤16mcg/mL = S
  Ciprofloxacin MIC: ≤1mcg/mL = S
  Doxycycline MIC: ≤1mcg/mL = S
  Imipenem MIC: ≤2mcg/mL = S
  Linezolid MIC: ≤1mcg/mL = S
  Moxifloxacin MIC: ≤0.5mcg/mL = S
  Tobramycin MIC: ≤2mcg/mL = S
  Trimethoprim/sulfamethoxazole MIC: 1/19 = S

Based on susceptibility data and resolution of symptoms after port removal and start of antimicrobial therapy, the patient was continued on levofloxacin and doxycycline totaling 6 weeks of therapy. A colonoscopy was performed about one month after completing the antibiotic course and revealed multiple benign tubular adenomas in the transverse colon. Our patient remains asymptomatic and free of signs of infection at 4 months after removal of his port and subclavian catheter.

## 3. Discussion

*Mycobacterium neoaurum* is a rapidly growing mycobacteria, part of the parafortuitum complex based on Runyon classification. It can be distinguished from other more common rapidly growing mycobacteria due to its smooth, round, and yellow-orange colonies [3]. Previously identified using a myriad of biochemical tests, more accurate identification systems for *M. neoaurum* currently use 16S ribosomal ribonucleic acid (16S rRNA) sequencing [4, 5].

*Mycobacterium neoaurum* like other rapidly growing NTM can cause a wide range of infections including skin and soft tissue, pulmonary, urinary tract, and catheter-related bloodstream infections [6]. M. *neoaurum* still remains an uncommon etiologic agent for bloodstream infections overall. Due to its ability to form biofilms, it has a predilection for prosthetic and indwelling devices. This also poses a challenge when treating the infection as biofilm formation makes it difficult to eradicate the nidus of the infection.

Patients most vulnerable to bacteremia from *M. neoaurum* or similar rapidly growing mycobacteria are often immunocompromised and receiving chemotherapy. The first case of human infection by *M. neoaurum* was in 1987 when it was isolated from the blood of an elderly female with metastatic cystadenocarcinoma of the ovary who had a Hickman catheter placed for total parenteral nutrition. She was successfully treated with seven weeks of gentamicin and cefoxitin [7]. Several case reports, one case series, and two large studies each including more than 150 patients with cancer and *M. neoaurum*, or other rapid-growing nontuberculous mycobacteria, bacteremia showed that most patients have a fever and had a central venous catheter [2, 8, 9].

In our case, the patient was considered free of previous colon cancer and had no known signs of immunocompromise. He did have multiple comorbid conditions including insulin-dependent type 2 diabetes, hypertension, remote history of seizure disorder, and schizophrenia with cognitive impairment which made it difficult to discern how much of his behavioral changes were due to the infection. He had also received several courses of antibiotics for urinary tract infections over the previous few months. Based on our review of the current literature, this is one of the few cases of port-related blood infection with *M. neoaurum* in a patient with a history of colon cancer but no known active malignancy. Drug susceptibility patterns vary greatly between rapidly growing mycobacteria species and optimal therapeutic regimens have not been established. No treatment guidelines are available, and treatment depends on the sensitivity results and the experience of the treating physician. Multiple regimens have been used with good response [10]. The *M. neoaurum* isolate from our patient had a susceptibility pattern consistent with most of the *M. neoaurum* isolates published in case reports and studies which indicated that most isolates were susceptible to aminoglycosides, clarithromycin, imipenem, trimethoprim-sulfamethoxazole, cefoxitin, tetracyclines, and fluroquinolones [4, 8, 11]. Our patient clinically improved after 6 weeks of therapy. He also had negative blood cultures after his port was removed and continues to be well.

## 4. Conclusion

Bloodstream infections due to rapidly growing mycobacteria like *M. neoaurum* are uncommon. Patients who are immunocompromised and possess implanted prosthetic devices and vascular access devices are at risk of developing these infections. Fortunately, device removal and appropriate antimicrobial therapy guided by susceptibility data are often enough to manage these infections.

## Data Availability

The data used to support the findings of this study are available from the corresponding author upon request.
